# Models Predicting Hospital Admission of Adult Patients Utilizing Prehospital Data: Systematic Review Using PROBAST and CHARMS

**DOI:** 10.2196/30022

**Published:** 2021-09-16

**Authors:** Ann Corneille Monahan, Sue S Feldman

**Affiliations:** 1 Department of Epidemiology & Public Health School of Public Health University College Cork Cork Ireland; 2 Department of Health Services Administration University of Alabama at Birmingham Birmingham, AL United States

**Keywords:** emergency services, hospital, decision support techniques, patient-specific modeling, crowding, boarding, exit block, systematic review, PROBAST, CHARMS, predictive model, medical informatics, health services research, prehospital assessment, process improvement, management information system, predict admission, emergency department

## Abstract

**Background:**

Emergency department boarding and hospital exit block are primary causes of emergency department crowding and have been conclusively associated with poor patient outcomes and major threats to patient safety. Boarding occurs when a patient is delayed or blocked from transitioning out of the emergency department because of dysfunctional transition or bed assignment processes. Predictive models for estimating the probability of an occurrence of this type could be useful in reducing or preventing emergency department boarding and hospital exit block, to reduce emergency department crowding.

**Objective:**

The aim of this study was to identify and appraise the predictive performance, predictor utility, model application, and model utility of hospital admission prediction models that utilized prehospital, adult patient data and aimed to address emergency department crowding.

**Methods:**

We searched multiple databases for studies, from inception to September 30, 2019, that evaluated models predicting adult patients’ imminent hospital admission, with prehospital patient data and regression analysis. We used PROBAST (Prediction Model Risk of Bias Assessment Tool) and CHARMS (Checklist for Critical Appraisal and Data Extraction for Systematic Reviews of Prediction Modeling Studies) to critically assess studies.

**Results:**

Potential biases were found in most studies, which suggested that each model’s predictive performance required further investigation. We found that select prehospital patient data contribute to the identification of patients requiring hospital admission. Biomarker predictors may add superior value and advantages to models. It is, however, important to note that no models had been integrated with an information system or workflow, operated independently as electronic devices, or operated in real time within the care environment. Several models could be used at the site-of-care in real time without digital devices, which would make them suitable for low-technology or no-electricity environments.

**Conclusions:**

There is incredible potential for prehospital admission prediction models to improve patient care and hospital operations. Patient data can be utilized to act as predictors and as data-driven, actionable tools to identify patients likely to require imminent hospital admission and reduce patient boarding and crowding in emergency departments. Prediction models can be used to justify earlier patient admission and care, to lower morbidity and mortality, and models that utilize biomarker predictors offer additional advantages.

## Introduction

### Background

The delivery of timely quality care in emergency departments has become increasingly challenging due to crowding [1,2]. Emergency department crowding is an international problem [3-5] that has been of continuing concern for the last two decades and is expected to become more problematic with population growth and an aging population whose life expectancy is increasing. The magnitude of the crowding problem has been demonstrated by decades of research into emergency department efficiency interventions that aimed to reduce crowding by improving throughput and processes, such as triage, diagnosis, and treatment, that affect the flow of care [6,7]. However, these measures primarily promoted efficiency in portions of the emergency department care continuum and had little effect in reducing crowding, because they did not address the source of the problem at a system level [8].

Rigorous analysis suggests that exit block and emergency department boarding are the main causes of emergency department crowding [6,9-12]. Boarding is the retention of patients who have already been admitted to the hospital in the emergency department because they await assignment to an inpatient hospital bed [5]. Exit block is the delay that occurs when patients cannot be transitioned into the hospital for admission or discharged (home, rehabilitation, etc) in a timely manner [5,8]. Exit block results in emergency department boarding and is a system issue [8,13]. Both boarding and the resulting overcrowding have been conclusively associated with poor patient outcomes and threats to patient safety [5,14-17].

### Predictive Modeling

Predictive modeling that can be used to address emergency department crowding is an emerging field of study. Predictive modeling is used to anticipate which factors will bring about a particular outcome [18]. In health care, models use specific data to estimate the probability that a condition or disease is already present (a diagnostic model) or the probability that an outcome will occur in the future (a prognostic model) [18]. Recent studies [19-28] of models utilizing these techniques estimate patient risk for health conditions and patient–provider encounters (eg, suicide attempts or intentional acts of self-harm) [19], acute kidney injury (ie, sudden kidney failure or damage) [20], hospital readmissions (ie, readmission to a hospital within 30 days of discharge, regardless of cause) [23,24,26,27], and perioperative mortality (ie, deaths within 30 days of surgery) [21], emergency department return visits (ie, return emergency department visits within 72 hours for any reason) [28], return visits after hospital discharge (ie, return emergency department visits within 30 days of hospital discharge for any reason) [25], and emergency department crowding or demand (ie, the availability of space for patients relative to the volume of patients that need to be seen) [22]) to improve health care delivery and patient outcomes. A subsection of this area of study focuses on predicting which emergency department patients are likely to require imminent hospital admission. This area of research is important because of its direct and immediate potential to lower patient morbidity and mortality by helping emergency department patients receive care earlier in the emergency department care continuum.

While more prediction models have been developed in recent years [18], external validation studies of published prediction models have not kept pace [29]. There is often no consensus about the best, most effective model for a particular purpose, leaving providers and policy makers unable to choose a model with confidence. In the case of hospital admission prediction, most models have not been externally validated or tested in a live emergency department environment. Furthermore, systematic reviews have received scrutiny for their lack of rigor [30-32]. Hence, a rigorous systematic review of studies of admission prediction models is needed to synthesize findings that researchers and decision-makers can rely on with confidence to address localized emergency department boarding, crowding, and exit block, as well as system-wide implications.

### Systematic Review Validation

Rigorous systematic reviews follow accepted approaches. PROBAST (Prediction Model Risk of Bias Assessment Tool) [33] can be used to identify potential sources of bias in individual prediction model studies, and CHARMS (Checklist for Critical Appraisal and Data Extraction for Systematic Reviews of Prediction Modelling Studies) [34] can also be used to identify potential sources of bias, organize information, and identify relevant information used to evaluate the prediction modeling studies. While the systematic review of clinical trials is generally a well-established field, the fields of health care prediction modeling and systematic review of such studies are not as well established, despite growth in these fields. For example, a search of Google Scholar for “systematic review” AND “prediction” AND “healthcare” demonstrated an increase of 410% in publications between decades (from n=45,900 in 2000-2010 to n=234,000 in 2010-2020). As the number of prediction modeling publications continue to grow, the need exists to apply the same rigor to systematic reviews of health care–related prediction modeling as that which has been applied to clinical trial and other types of systematic reviews through the use of tools, such as PROBAST and CHARMS, to facilitate quality assessment for individual prediction model studies using standardized guidelines [30,33]. Only two systematic reviews [35,36] that have focused on increasing overall throughput by decreasing emergency department boarding and systemic exit block in health systems applied the rigorous PROBAST and CHARMS methodologies, with both reporting a high degree of bias in the studies that they examined.

### Logistic Regression for Systematic Reviews

Logistic regression is a technique for understanding the relationships between predictor variables and outcomes and is one of the most commonly used methods for forecasting [37]. There are a variety of techniques that can be used to model data; each is designed to accommodate types of data, number of predictors, and study aims, and each has advantages and disadvantages. Logistic regression is only used for data with a binary outcome and multiple predictors and accommodates predictors of multiple data types, such as continuous and categorical data; therefore, data types do not need to be modified, which can introduce potential bias. Logistic regression produces a mathematical form—a weighted combination of variables that predict the outcome variable [37].

We aimed to better understanding predictive modeling’s role in addressing the emergency department crowding problem by examining model predictive performance, the utility of the contribution of prehospital patient data to model prediction, applications of models, and the utility of models.

## Methods

### Study Design

We applied PROBAST and CHARMS to rigorously assess studies of models designed to predict adult patient imminent hospital admission using prehospital patient data collected early in the emergency department visit or during ambulance transport to the emergency department. We searched databases for papers published from inception through September 30, 2019. Data were organized and analyzed in Excel (version 2016, Microsoft Inc). This study did not require institutional review board authorization.

### Data Sources and Search Strategy

We reviewed database content descriptions for 99 health science, public health, and medical databases to determine their relevance to our topic of interest, and 13 databases were found to be relevant: EBSCO Database (includes Medline database and Academic Search Complete database), CINAHL Plus with Full Text, Cochrane Library, Health and Safety Review, ProQuest Central, Scopus, BMJ Journals, JAMA, Journals at Ovid, PLOS, SAGE Journals, ScienceDirect, and NIHR/PROSPERO.

**Figure 1:**
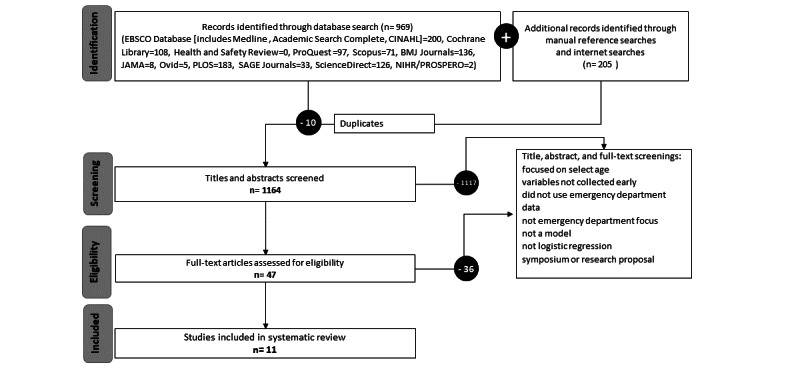
Search flow diagram of included studies.

The *Title, abstract, or keyword* option was used with the following search string: “model or strategy and hospital* and predict* or risk.” (Asterisks were used to capture hospital, hospitalization, hospitalisation, hospitalized, hospitalized and predict, predicts, predicted, predictor, predictive.) If no results were initially produced, the search was expanded by removing all filters and searching for the terms anywhere in the document. Sources that did not allow for truncation were searched multiple times with multiple word combinations. Additionally, the internet was searched with the following combined terms: “model predict hospital admission,” “risk of hospital admission,” “hospital admission model,” “admission risk,” “emergency model,” and “hospital admission.” Reference lists were also reviewed (Figure 1).

### Inclusion and Exclusion Criteria

We included full-text peer-reviewed English-language studies that evaluated strategies or models using prehospital patient data to predict imminent hospital admission of primarily adult general medicine patients with regression.

Studies in which the setting was not an emergency department, data were not collected early in the emergency department visit, or either models or logistic regression were not used and that focused on pediatric (<16 years of age), psychiatric, or specific health conditions were excluded.

### Data Quality Assessment

We used PROBAST to assess risk of bias for each study. Shortcomings in a study’s design, conduct, or analysis can cause systematic errors that result in flawed or distorted results and hamper internal validity [18]. Assessment of the quality of studies, including risk of bias and model applicability to the target settings and populations, is an essential component of systematic reviews and their evidence synthesis. The first step in applying PROBAST was the identification of a clear and focused review question about the intended use of the model, targeted participants, predictors used in the modeling, and predicted outcome [33]. The second step was the identification and assessment of potential sources of bias in 4 domains (participants, predictors, outcomes, analysis). Key qualities assessed for each study included the appropriateness of the data source, whether predictors were similarly measured and defined, whether outcomes were measured similarly for all participants, and whether missing data were appropriately handled and reported.

### Data Extraction and Data Synthesis

We used CHARMS to identify key items in 11 domains (eg, source of data, sample size, model development, model performance, results) in individual studies (and in their PROBAST reports) in order to evaluate potential sources of bias and issues that may affect the applicability of results in relation to the intended use of the model. Key information was organized by relevant domains (Multimedia Appendix 1).

## Results

### General

Searches produced 1164 citations, from which 47 were selected for full review; 11 studies met inclusion criteria. Each model was critically assessed with PROBAST (Multimedia Appendix 2) and CHARMS.

### CHARMS Study Characteristics

#### Data Source, Participants, and Outcome CHARMS Domains 1, 2, and 3

Of the 11 studies, 3 used a prospective observational cohort [38-40], and the remaining 8 used a retroactive observational cohort [22,41-47]. There was good diversity, in terms of the countries in which studies took place (South Africa [38], Scotland [41], the United States [22,42,44,45], the Netherlands [40,43], Australia [39], and Singapore [46,47]). Sampling ranged from 14 days [40] to 10 years [46], with most study durations between 3 and 27 months [38,39,41-43,45,47]. Two studies were 2 months in length [22,44].

Most studies utilized clinical and administrative patient information collected early in the emergency visit [22,38-43,46,47]; 2 studies used data collected during ambulance transport to the emergency department [44,45]. Additionally, all studies evaluated 1 or more models’ abilities to predict patient imminent need for hospital admission and defined outcome event by patient final disposition, and measured outcome by patient hospital admission or discharge from the emergency department. Furthermore, all studies corresponded to the outcome definition of the systematic review question, which reduced the potential for bias from different outcome definitions and measurement methods that can lead to differences in study results and would be a source of heterogeneity across studies [34].

#### Candidate Predictors CHARMS Domain 4

Candidate predictors included all predictors investigated in a given study for predictive performance and not the finalized predictors included in model analysis. Candidate predictors ranged from 5 to 14 per study (Multimedia Appendix 3): under 10 predictors [22,38,39,45], over 10 predictors [40,41,43,44,46,47], and did not report [42]. Overall, 52 candidate predictors had been evaluated, and 34 predictors were retained in models (across all studies).

#### Sample Size CHARMS Domain 5

Consideration of sample size is important to ensure adequate numbers of data events are collected to achieve meaningful results. Sample sizes ranged from 401 to 864,246. None reported sample size calculation, estimation, or rationale. One study [40] did, however, perform a sample size calculation for its validation. All studies described efforts to avoid overfitting, which included model comparison to validation models [22,38,40,41,43,44,46,47], model comparison to multiple site outcomes [45], model comparison to published models [42], and model comparison to triage nurse prediction of patient final disposition [39]. Overfitting describes when findings in the development sample do not exist in the relevant population resulting in a model that too closely fits the development data set and produces findings that are not reproducible [37]. Overfitting is a primary concern in prediction modeling development that can be mitigated by performing sample size estimates during study design [34].

#### Missing Data CHARMS Domain 6

Infrequently is value attributed to missing data in the missing state [48]; instead, the missing values are either imputed or disregarded completely [49,50]. Four studies described a process for handling missing data: 3 used multiple imputation [39,41,43], and 1 study reported “missing predictors were replaced with missing values” [42]; it was unknown whether this referred to blank (ie, missing) identifiers or whether missing values were imputed. Of the remaining 7 studies, 1 study reported 30% of data were missing and did not describe how missing data were handled (ie, whether the patient events were included or excluded) [38], and 6 studies did not mention missing data at all [22,40,44-47].

#### Model Development CHARMS Domain 7

Two studies also developed models using other techniques (gradient boosting and deep neural network [42], and naive Bayes [22]) in addition to models using logistic regression. Most studies selected predictors using univariate analysis [22,39,40,42,43,46,47], but 4 studies used multivariate modeling [38,41,44,45].

#### Model Performance CHARMS Domain 8

Model predictive performance was gauged via the percentage of patients actually admitted, the percentage of patients predicted to be admitted, and goodness of fit tests that assessed model discrimination and model calibration (Table 1).

**Table 1:** Model performance predicting patient hospital admission.

Reference	Model performance			
	Admission		Goodness of fit tests	
	Actual, n (%)	Predicted, %	Discrimination, AUROC^a^ (95% CI)	Calibration^b^
Burch et al [38]	469 (59)	—^c^	—	—
Cameron et al [41]	—	—	0.88 (0.88-0.88)	—
Hong et al [42]	60,277 (29.7)	—	0.86(0.86-0.87)	—
Kim et al [39]	38,695 (38.6)	—	0.80 (0.80-0.80)	Performed, not reported
Kraaijvanger et al [40]	400 (31.7)	31.1	0.87 (0.85-0.89)	Reported to be good
Lucke et al [43]	2912 (27)	21.4	0.86 (0.85-0.87)	Reported to be good
Meisel et al [44]	132 (33)	32	0.80 (—)	Performed, not reported
Meisel et al [45]	440 (24.8)	39.8	0.83 (—)	—
Parker et al [46]	334,115 (38.7)	—	0.83 (0.82-0.83)	Reported to be good
Peck et al [22]	—	—	0.89 (—)	r^2^=0.58 moderate to poor
Sun et al [47]	95,909 (30.2)	30	0.85 (0.85-0.85)	Reported to be good

^a^AUROC: area under the receiver operating characteristics curve.

^b^Studies used several formulas to evaluate calibration, to include Hosmer-Lemeshow, threshold probability, and r^2^.

^c^Not reported.

Discrimination is a model’s ability to distinguish between patients who do and do not experience the outcome of interest and is most commonly assessed with the area under the receiver operating characteristics (AUROC) [51]. The AUROC represents the performance of a classification model that has a categorical outcome, producing a score representing a proportion of times the model correctly discriminated between groups, for example, those at high risk and low risk. The higher the AUROC, the better the model discriminates between the 2 groups (0.5-0.6 represents not better than chance, 0.6-0.7 represents poor, 0.7-0.8 represents fair, 0.8-0.9 represents good, and 0.9-1.0 represents excellent discrimination [52]). Eight studies reported good discrimination [22,40,47], 2 reported fair discrimination [39,53], and 1 study did not report any performance measurement [38].

Calibration is the extent to which model predicted risk compares to observed outcomes (ie, difference between rates of observed events and predicted events for groups [54]. Calibration is usually reported graphically by plotting observed against predicted event rates [55] and is commonly measured with the Hosmer-Lemeshow statistical test for binary categorical outcomes [54]. Most studies that measured calibration statistically, reported good agreement between predicted and observed hospital admission. Seven models evaluated calibration using Hosmer-Lemeshow [44,47,43,39], threshold probability of admission [46], or R^2^ [22], 1 did not report which statistic was used [40], and 2 of these 7 studies did not report results [39,44]. Four studies did not measure calibration [38,41,42,45].

#### Model Evaluation: Domain 9

Utility of predictive models depends on their external validation—performance evaluation on an independent data set. External validation took a variety of forms: different settings with different samples [40], same locations with different samples [43,45,46], and nurse opinion on likely patient admission [22,39]. Five models were internally validated [38,41,42,44,47].

#### Model Results: Domain 10

Predictive accuracy and precision drive model performance and the extent to which it can estimate the probability of individual patient outcomes, as well as model suitability for clinical and administrative uses.

The models in the 11 studies were not operational (no apps developed and no integration with information systems or workflow) and were not tested in environments in which they would be used, which compromised the evaluation of model feasibility. Operational models would identify patients likely to require hospital admission; thus, there is a great amount of utility and potential for models to improve patient care and hospital operations, including by reducing hospital exit block, emergency department boarding, and ultimately emergency department crowding.

#### Interpretation and Discussion: Domain 11

The utility of select prehospital patient data to act as predictors and as data-driven, actionable tools to identify patients requiring hospital admission was shown. The models utilizing biomarker predictors (eg, blood pressure, heart rate) [38,43,45] may provide advantages due to standardized definition, measurement, and interpretation of these biomarker measures. Models that use only biomarker predictors may be widely applicable and robust, and their results may be generalizable to populations and environments. Models that did not include patient history variables (eg, chronic conditions, number of prior emergency department visits) [22,38,40,47] may have greater applicability because the model does not rely on the availability of medical record information or patient reports. The predictors in these models—prehospital patient data collected early in the emergency department visit or during ambulance transport—are not the only options for predicting patient admission but are likely the best options for making timely predictions using data collected in the early stages of an urgent care visit.

AUROC values suggested fair to good ability to distinguish between outcome groups (admitted, not admitted), and thus, to predict patient imminent need for hospital admission. Likewise, the utility of the variables as predictors for the identification of patients likely to require imminent hospital admission was shown.

#### Risk of Bias Assessment

Data transformation can increase risk of bias by satisfying assumptions without changing the scale of representation [56]. Five studies did not transform raw data [38,44-47]. On the other hand, 6 studies transformed predictors, such as, by categorizing continuous variables and dichotomizing continuous variables [22,39-43].

Evaluation of heterogeneous predictors across studies introduces bias if they are treated as identical. In 2 studies, bias was low, because standardized, frequently calibrated equipment was used to measure predictors (eg, blood pressure, laboratory analysis, etc), which produces measurements that are comparable across studies, required no manipulation (eg, dichotomized, categorized), and offer more likelihood of retaining reliability when applied to new populations [38,43]. Age has been shown to inject bias, for example, the same model can appear to perform better when applied to a sample with a wide age range than when applied to a sample with a narrow age range [57]. Nine models included age [22,39-41,43-47], with only 2 studies indicating age >60 years [44,45].

Estimating sample size during study design minimizes model overfitting and includes calculating events-per-variable. Events-per-variable, generally, is poorly reported in prediction model studies [34] and was not reported in any of the included studies. However, events-per-variable can be calculated from other study information to aid assessment of study quality. The appropriateness of most studies’ sample size could be evaluated by calculating study events-per-variable, the number of data events needed per predictor variable to achieve meaningful results [37]. This ratio was calculated using study limiting sample size, the portion of outcome events (admitted or not admitted) that is smaller [37]. The focus is on the smaller portion of outcome events, because the total sample size is not directly relevant in binary models [37]. The limiting sample size is divided by the number of candidate predictors to produce the limiting events-per-variable ratio.

In 10 studies [22,39-47], the limiting sample size was the number of admitted patients, but in 1 study [38] the limiting sample size was the number of patients who were not admitted (ie, more patients were admitted than discharged). Limiting events-per-variable could not be calculated for 3 models because either the proportion of admitted patients or the number of candidate predictors was not reported [22,41,42]. The limiting sample size range of studies was 132.3 to 334,115, producing a limiting events-per-variable range of 9 to 30,374. The limiting events-per-variable was sufficient in most studies to obtain meaningful results and avoid bias from an overfitted model. However, at 9 events-per-variable, 1 model [44] was below the recommended 10 to 15 events-per-variable [42,58,59] and was in jeopardy of bias.

Missing data handling can inject bias. To mitigate against bias with imputation, 3 studies used multiple imputation [39,41,43], substituting missing observations with plausible estimated values derived from analysis of available data, which is the preferred method for handling missing data in prediction research [34,60]. One study [42] reported replacing missing values but did not disclose how these missing values were placed, and the remaining 7 studies did not describe the handling of missing data [22,38,40,44-47], which suggested there was an element of risk of bias. Data are usually not missing at random and instead are related to other observed participant data and, as a consequence, participants with complete data are different from those with incomplete data [34,61].

Per PROBAST definition, a model that is internally validated is a development-only study—not a development and validation study. A model must be externally validated to be considered a development and validation study. While 6 of the models were externally validated [22,39,40,43,45,46], 2 studies used nurses’ opinions [22,39] and were not validated with data.

Inclusion of false predictors increases the likelihood of model overfitting because the model corresponds too closely to its derivation data set and fails to fit other relevant data sets or predict future observations reliably [62], resulting in overly optimistic predictions of model performance for new data sets [34]. In univariate analysis, each predictor is tested individually for its association with the outcome, and the most statistically significant predictors are included in the model. However, univariate analysis is not the preferred method because it commonly introduces selection bias when predictors selected for model inclusion have a large but false association with the outcome [18,63]. In small samples, predictors could initially show no association with outcome, but after adjustment for other predictors, may show association with the outcome [34]. Conversely, multivariate modeling is preferred for predictor selection because there is no selection bias since all predictors are prespecified. Only 4 of the models used multivariate modeling for predictor selection [38,41,44,45], and the remaining models used univariate analysis [22,39,40,42,43,46,47].

## Discussion

### Principal Findings

This study showed the utility of select, prehospital patient data to act as predictors to model identification of patients likely to require hospital admission and that models produced information that could be used to improve patient care and hospital operations. Ten studies reported model discrimination with AUROC: 8 studies reported values [22,40-43,45-47] that suggest good ability to distinguish between outcome groups (admitted, not admitted), and thus, to predict patients’ imminent need for hospital admission. An example of model application for patients who are predicted to require admission is earlier bed request giving managers more time to secure a patient bed. This forewarning could result in operations procedures to decrease exit block and increase patient flow out of the emergency department [13].

Potential sources of bias that may cause flawed or distorted model predictions were found in every model, for example, from minor (not reporting handling of missing values [38,39,43,44,47], univariate predictor selection [39,47]) to potentially damaging (dichotomized continuous variables [22,41,43], low events-per-variable [44], no external validation [38,41,42,44,47]), which suggest that study reports of models’ abilities to predict outcomes have the potential to be flawed. This is consistent with other evaluations of prediction modeling studies [34], including evaluations applying CHARMS and PROBAST in the emergency department setting [35,36].

Overall, model performances were reportedly good, with most models showing good ability to discriminate between patients who do and do not require imminent hospital admission [22,40-43,45-47], and almost half reporting good calibration to detect differences between observed and predicted admission rates [40,43,46,47]. Although several studies did not measure calibration [38,41,42,45], the remainder did [22,39,40,43,44,46,47]. However, all [38-47] but 1 study [22] poorly reported its measurement. Findings of neglected calibration measures, with an overreliance on discrimination measures, are consistent with those of other reports [34]. Assessing and reporting discrimination and calibration are important in prediction model evaluation. No models were found to have operated through an app, and none had been integrated with an information system. However, to function as intended, most models required development of an electronic app to receive patient data, operate the algorithm, and produce results. Most also required app integration with an information system to produce real-time admission prediction. Studies also did not describe a process to achieve app development or system integration.

Biomarker predictors may contribute superior value and advantage to a model due to their lack of variability in definition, measurement, and interpretation, and freedom from the confines of patient histories, resulting in a widely applicability.

The quantity of candidate predictors demonstrated the breadth of potential influences on patients’ imminent need for hospital admission. However, the number of predictors across studies did not reflect the quantity accurately because, across studies, multiple names were used for the same predictor—identically named predictors were defined differently, data collection and evaluation varied, and predictors composed of multiple variables were not specified

Models have the potential to facilitate hospital admission, subsequently reducing or ending hospital exit block, emergency department boarding, and emergency department crowding but none had been implemented or tested.

To develop models with the most potential, future investigations must address deficiencies, avoid risk of bias in model design and investigation, verify the utility of biomarker predictors and the most useful predictor combination, evaluate real-time utility of admission prediction on hospital operations, compare performance of technology enabled versus intuition, and verify longitudinal model impact on patient care and hospital operations.

### Limitations

Although the findings of this review are valuable and add to the current literature on artificial intelligence models in the emergency department setting, this study has several limitations. First, this was a critique of the methodologies used in the models; we did not consider the feasibility of the models examined. Second, the selection of studies and PROBAST assessments were performed by one researcher, with a second researcher providing oversight. The use of multiple researchers would have ensured intercoder reliability and mitigated systematic errors. Additionally, only studies in English and conducted with emergency department setting data were included. That being said, this study closely adhered to the CHARMS methodology for study evaluation.

### Comparison With Prior Work

We applied both CHARMS and PROBAST to studies that used logistic regression and data from emergency department settings. Our findings are consistent with those of previous systematic reviews [35,36,64,65] that applied PROBAST and CHARMS methodologies to evaluate health care prediction models, in terms of risk of bias. We attempted to be focused and provide depth of analysis by identifying and appraising hospital admission prediction models that utilized prehospital patient data in a defined setting (emergency department). Four healthcare prediction model studies were reviewed for their use of PROBAST and CHARMS methodologies. However, while 2 [35,36] were set in the emergency department, evaluation variables and outcome of interest differed for all 4 studies [35,36,64,65].

## Appendix

Multimedia Appendix 1 Study characteristics by CHARMS domains.

Multimedia Appendix 2 Completed PROBAST.

Multimedia Appendix 3 Predictors evaluated by each study.

## Abbreviations

AUROC: area under the receiver operating characteristics curve
CHARMS: Checklist for Critical Appraisal and Data Extraction for Systematic Reviews of Prediction Modelling Studies
PROBAST: Prediction Model Risk of Bias Assessment Tool
