# Supplementary material for: Models Predicting Hospital Admission of Adult Patients Utilizing Prehospital Data: Systematic Review Using PROBAST and CHARMS
Source: JMIR Med Inform. 2021 Sep 16;9(9):e30022. doi: 10.2196/30022 (PMC8485197; doi:10.2196/30022)
Supplement: Multimedia Appendix 1 [file medinform_v9i9e30022_app1.docx]

Appendix 1. Study characteristics organized by CHARMS domains 1-11 and domains features

|  | **D1: Source of Data** | | **D2: Participants** | | | **D3: Outcome** | **D4: Candidate Predictors** | | **D5: Sample Size** | | | | **D6: Missing Data** | **D7: Development** | | | **D8: Performance** | | **D9: Evaluation** | **D10: Results** | **D11: Interpretation and Discussion** |
| --- | --- | --- | --- | --- | --- | --- | --- | --- | --- | --- | --- | --- | --- | --- | --- | --- | --- | --- | --- | --- | --- |
| **Author(s) and date** | Cohort Design | Data source | Population | Country | Sampling | Outcome | # candidate predictors | # and final predictors | Sample size | Admitted n(%) | Limiting EPV^7^ | Overfit avoidance | % missing and how handled | Study type | Modeling technique | Predictor selection | Admission:  Actual  predicted | Goodness fit: Discrimination1  Calibration^2^ | Validation | a. Predictive performance,  b. Predictor utility,  c. Model application,  d. Utility (if operational) | overall findings, per systematic review objectives |
| **Burch et al, 2008** **[38]** | PO3 | P4 | ED patients,  adult general medicine | South Africa | 3 m5 | A/D6 | 5 | 5: Systolic blood pressure ≤ 100 mm Hg, pulse rate ≥ 130 beats per minute, respiratory rate ≥ 30 breaths per minute, temperature ≥ 38.5C, impaired level of consciousness | 790 | 469 (59%) | 64 | NSSC8  Comparison to validation model | 30% data missing, no mention of how handled | D^9^ | ^LR10^ | MVM11 | 59%  N/R1^2^ | N/R  N/R | Internal:model comparison | a. uncertain due to ROB. b. 5/5 were predictive.  c. yes, in current state, real-time, by-hand.  d. ID patients likely to require hospital admission. | Utility of prehospital patient data contributed to identification of patients requiring hospital admission.  Models utilizing biomarkers may provide advantages due to standardization in definition, measurement, and interpretation.  Models excluding prior patient data would be expected to improve model application.  Inconsistencies in a predictors’ contributions to the model and discovery of a best predictor combination are a primary reason for further investigation.  Further investigation of in situ application of the model to realize the true predictive value these models could offer. |
| **Cameron et al, 2015 [41]** | RO^3^ | D | ED patients,  adult general medicine | Scotland | 2 y^5^ | A/D | 10 | 6: Acuity category/score (MTS and NEWS), age, ambulance arrival, referral source, admission within the last year | 215,231 | N/R | N/R | NSSC.  Comparison to validation model | Multiple imputation | D | LR | MVM | N/R  N/R | 0.88 (95% CI: 0.88-0.88)  N/R | Internal: model comparison | a. uncertain due to ROB.  b. 6/10 were predictive.  c. yes with tech integration. But could apply by-hand, real-time.  d. ID patients likely to require hospital admission. |  |
| **Hong et al [42]** | RO | D | ED patients,  adult general medicine | US | 1 y | A/D | N/R | 4: ESI level, outpatient medication counts, demographics, hospital usage statistics | 202,953 | 60,277 (29%) | N/R | NSSC.  Model compared to other models | Missing predictors replaced with missing values, but unknown how | D | LR | UVA^11^ | 29.7%  N/R | 0.86- (95% CI: 0.86-0.87)  N/R | Internal:  random split sample | a. uncertain due to ROB.  b. 4/4 were predictive.  c. no, no info on prediction score.  d. ID patients likely to require imminent hospital admission. |  |
| **Kim et al, 2014 [39]** | PO | D | ED patients,  adult general medicine | Australia | 2 y 3 m | A/D | 7 | 7: Age, presenting symptom or diagnosis, Australasian Triage Scale (ATS) category, ambulance arrival, outside referral, triage time of day, day of week | 100,123 | 38,695 (38.6%) | 5,528 | NSSC.  Model compared to triage nurse prediction | Multiple imputation | D,  V^9^ | LR | UVA | 38.6%  N/R | 0.80 (95% CI: 0.80-0.80)  N/R | External: nurse opinion on likely patient admissions | a. uncertain due to ROB.  b. 7/NR were predictive.  c. yes, with tech integration.  d. ID patients likely to require imminent hospital admission. |  |
| **Kraaijvanger et al, 2018 [40]** | PO | D | ED patients,  adult general medicine | Netherlands | 14 d^5^ | A/D | 10 | 4: Age, triage category, arrival mode, main symptom | 1,261 | 400 (31.7%) | 40 | NSSC Comparison to validation model | No mention of missing data | D,  V | LR | UVA | 31.7%  31.1% | 0.87 (95% CI: 0.85-0.89)  good | External: different setting, different sample | a. uncertain due to ROB.  b. 4/10 were predictive.  c. yes, with tech integration.  d. version of it operational. ID patients likely to require imminent hospital admission. |  |
| **Lucke et al, 2018 [43]]** | RO | D | ED patients,  adult general medicine | Netherlands | 5 m | A/D | 13 | 13: Age, sex, acuity category, mode of arrival, blood tests ordered, chief complaint, revisit ED within 30 days, type of specialist (patient is medicine or surgery), phlebotomised blood sample taken, blood pressure, pulse rate, respiration, temperature | 10,807 | 2,912 (27%) | 224 | NSSC.  Comparison to validation model | Multiple imputation | D,  V | LR | UVA | 27%  21.4% | 0.86 (95% CI: 0.85-0.87  good | External: same location w/ different sample | a. uncertain due to ROB.  b. 13/13 were predictive.  c. yes, with tech integration.  d. ID patients likely to require imminent hospital admission. |  |
| **Meisel et al, 2008 [44]** | RO | D | Ambulance patients,  adult general medicine | USA | 2 m | A/D | 14 | 6: Dyspnea, chest pain, dizziness/weakness/syncope, age ≥60, history of diabetes, history of cancer | 401 | 132 (33%) | 9 | NSSC.  Comparison to validation model | No mention of missing data | D | LR | MVM | 33%  32% | 0.80 (-)  N/R | Internal: bootstrapping with replacement | a. uncertain due to ROB.  b. 6/14 were predictive.  c. yes, in current state, real-time, by-hand.  d. ID patients likely to require imminent hospital admission. |  |
| **Meisel et al, 2009 [45]** | RO | D | Ambulance patients,  adult general medicine | USA | 1 y | A/D | 6 | 6: age ≥60 years, chest pain, shortness of breath, dizziness/weakness/syncope, history of cancer, history of diabetes | 1,102 | 440 (40%) | 73 | NSSC.  Model compared to multiple sites | No mention of missing data. | V | LR | MVM | 24.8%  39.8% | 0.83 (-)  N/R | External: same location with different sample | a. uncertain due to ROB.  b. 6/6 were predictive.  c. yes, in current state, real-time, by-hand.  d. ID patients likely to require imminent hospital admission. |  |
| **Parker et al, 2018 [46]** | RO | D | ED patients,  adult general medicine | Singapore | 10 ys | A/D | 11 | 8: Age, race, postal code, day of week, time of day, acuity category (PACS), mode of arrival, fever status | 864,246 | 334,115 (38.7%) | 30,374 | NSSC.  Comparison to validation model | No mention of missing data | D,  V | LR | UVA | 38.7  N/R | 0.83  0.82-.83  good | External: same location with different sample | a. uncertain due to ROB.  b. 8/11 were predictive.  c. yes, with tech integration.  d. ID patients likely to require imminent hospital admission. |  |
| **Peck et al, 2012 [22]** | RO | D | ED patients,  adult general medicine | USA | 2 m | A/D | 6 | 4: Age, primary complaint, bed type designation, arrival mode | 1,160 | N/R | N/R | NSSC.  Comparison to validation model. | No mention of missing data. | D,  V | LR | UVA | N/R  N/R | 0.89 (-)  r^2^ of 0.58 moderate to poor | External: nurse opinion on likely patient admissions | a. uncertain due to ROB.  b. 4/6 were predictive.  c. nos, req statistical development, tech integration.  d. ID patients likely to require imminent hospital admission. |  |
| **Sun et al, 2011 [47]** | RO | D | ED patients,  all ages general medicine | Singapore | 1 y | A/D | 10 | 3: Age, acuity score (PAC), arrival mode | 317,581 | 95,909 (30.2%) | 9,591 | NSSC.  Comparison to validation model. | No mention of missing data. | D | LR | UVA | 30.2%  30% | 0.85(95% CI: 0.85-0.85)  good | Internal: random split sample | a. uncertain due to ROB.  b. 3/10 were predictive.  c. yes, with tech integration.  d. ID patients likely to require imminent hospital admission. |  |
| 1 All studies that measured discrimination used AUROC. The higher the AUROC score the better the model discriminates between the 2 groups: 0.5-0.6=not better than chance; 0.6-0.7=poor; 0.7-0.8=fair; 0.8-0.9=good; 0.9-1.0=excellent.  2 Studies used several formulas to evaluate calibration.  3 PO=Prospective Observational or RO= Retrospective Observational  4 P=paper files or D=digital files  5 y=year or m=month or d=day  6 A/D=admitted or discharged  7 EPV=Events per Variable  ^8^NSSC=no sample size calculation  ^9^ D=development or V=validation  ^10^ LR=logistic regression  1^1^ MVM=multivariate modeling or UVA=univariate analysis  1^2^ N/R=not reported | | | | | | | | | | | | | | | | | | | | | |
