# Supplementary material for: Models Predicting Hospital Admission of Adult Patients Utilizing Prehospital Data: Systematic Review Using PROBAST and CHARMS
Source: JMIR Med Inform. 2021 Sep 16;9(9):e30022. doi: 10.2196/30022 (PMC8485197; doi:10.2196/30022)
Supplement: Multimedia Appendix 2 [file medinform_v9i9e30022_app2.docx]

## Appendix 2: Completed PROBAST

## PROBAST

(Prediction model study Risk Of Bias Assessment Tool)

Published in Annals of Internal Medicine (freely available):

1. [PROBAST: A Tool to Assess the Risk of Bias and Applicability of Prediction Model Studies](https://annals.org/aim/fullarticle/2719961/probast-tool-assess-risk-bias-applicability-prediction-model-studies)
2. [PROBAST: A Tool to Assess Risk of Bias and Applicability of Prediction Model Studies: Explanation and Elaboration](https://annals.org/aim/fullarticle/2719962/probast-tool-assess-risk-bias-applicability-prediction-model-studies-explanation)

| **What does PROBAST assess?**  PROBAST assesses both the *risk of bias* and *concerns regarding applicability* of a study that evaluates (develops, validates or updates) a multivariable diagnostic or prognostic prediction model. It is designed to assess primary studies included in a systematic review.  *Bias* occurs if systematic flaws or limitations in the design, conduct or analysis of a primary study distort the results. For the purpose of prediction modelling studies, we have defined *risk of bias* to occur when shortcomings in the study design, conduct or analysis lead to systematically distorted estimates of a model’s predictive performance or to an inadequate model to address the research question. Model predictive performance is typically evaluated using calibration, discrimination and sometimes classification measures, and these are likely inaccurately estimated in studies with high risk of bias. *Applicability* refers to the extent to which the prediction model from the primary study matches your systematic review question, for example in terms of the participants, predictors or outcome of interest.  A primary study may include the development and/or validation or update of more than one prediction model. A PROBAST assessment should be completed for each distinct model that is developed, validated or updated (extended) for making individualised predictions. Where a publication assesses multiple prediction models, only complete a PROBAST assessment for those models that meet the inclusion criteria for your systematic review. Please note that subsequent use of the term “model” includes derivatives of models, such as simplified risk scores, nomograms, or recalibrations of models.  PROBAST is not designed for all multivariable diagnostic or prognostic studies. For example, studies using multivariable models to identify predictors associated with an outcome but not attempting to develop a model for making individualised predictions are not covered by PROBAST.  PROBAST includes four steps.   \| **Step** \| **Task** \| **When to complete** \| \| --- \| --- \| --- \| \| **1** \| Specify your systematic review question(s) \| Once per systematic review \| \| **2** \| Classify the type of prediction model evaluation \| Once for each model of interest in each publication being assessed, for each relevant outcome \| \| **3** \| Assess risk of bias and applicability \| Once for each development and validation of each distinct prediction model in a publication \| \| **4** \| Overall judgment \| Once for each development and validation of each distinct prediction model in a publication \|   If this is your first time using PROBAST, we strongly recommend reading the detailed explanation and elaboration (E&E, see link above) paper and to check the examples on www.probast.org |
| --- | --- | --- | --- | --- | --- | --- | --- | --- | --- | --- | --- | --- | --- | --- | --- |

**Step 1: Specify your systematic review question**

| State your systematic review question to facilitate the assessment of the applicability of the evaluated models to your question. *The following table should be completed once per systematic review.* |
| --- |

| **Criteria** | **Specify your systematic review question** |
| --- | --- |
| *Intended use of model:* | To identify and critically appraise models designed to predict adult patient imminent hospital admission using pre-hospital patient data by examining: model predictive performance, utility of pre-hospital patient data to contribute to model prediction, applications of the models, and utility of the models. |
| ***Participants*** *including selection criteria and setting:* | full-text; peer-reviewed; English-language studies that evaluated strategies or models that used pre-hospital patient data to predict imminent hospital admission of primarily adult, general medicine patients; and examined data relationships with regression analysis^[[1]](#footnote-1)^. Mixed population (adult, pediatric, psychiatric) studies were included if the proportion of pediatric and psychiatric patients was small. Studies focused on pediatric (i.e., <16), psychiatric, or specific health conditions were excluded because: pediatric patient admission threshold is lower than adult admission threshold; psychiatric and specific health condition patient symptomology and clinical variables evaluated to determine course of treatment are significantly clinically different from a general medicine population. Date and source of publication were not considered. |
| ***Predictors*** *(used in prediction modelling), including types of predictors (e.g. history, clinical examination, biochemical markers, imaging tests), time of measurement, specific measurement issues (e.g., any requirements/ prohibitions for specialized equipment):* | administrative and clinical patient information collected early in the emergency care visit |
| *Outcome to be predicted:* | *Hospital admission* |

**Step 2: Classify the type of prediction model evaluation**

| Use the following table to classify the evaluation as model development, model validation or model update, or combination. Different signalling questions apply for different types of prediction model evaluation. If the evaluation does not fit one of these classifications then PROBAST should not be used. |
| --- |

| **Classify the evaluation based on its aim** | | | |
| --- | --- | --- | --- |
| **Type of prediction study** | **PROBAST boxes to complete** | **Tick as appropriate** | **Definition for type of prediction model study** |
| Development only | Development | ✓ | Prediction model development without external validation. These studies may include internal validation methods, such as bootstrapping and cross-validation techniques. |
| Development and validation | Development and validation |  | Prediction model development combined with external validation in other participants in the same article. |
| Validation only | Validation |  | External validation of existing (previously developed) model in other participants. |

| *This table should be completed once for each publication being assessed and for each relevant outcome in your review.* | |  |
| --- | --- | --- |
| **Publication reference** | Cameron, A., Rodgers, K., Ireland, A., Jamdar, R., & McKay, G.A., (2015). A simple tool to predict admission at the time of triage. Emerg Med J. Mar, 32(3):174-9. | |
| **Models of interest** | Prediction model | |
| **Outcome of interest** | Hospital admission | |

**Step 3: Assess risk of bias and applicability**

| PROBAST is structured as four key domains. Each domain is judged for risk of bias (low, high or unclear) and includes signalling questions to help make judgements. Signalling questions are rated as yes (Y), probably yes (PY), probably no (PN), no (N) or no information (NI). All signalling questions are phrased so that “yes” indicates absence of bias. Any signalling question rated as “no” or “probably no” flags the potential for bias; you will need to use your judgement to determine whether the domain should be rated as “high”, “low” or “unclear” risk of bias. The guidance document contains further instructions and examples on rating signalling questions and risk of bias for each domain.  The first three domains are also rated for concerns regarding applicability (low/ high/ unclear) to your review question defined above.  *Complete all domains separately for each evaluation of a distinct model. Shaded boxes indicate where signalling questions do not apply and should not be answered.* |
| --- |

| **DOMAIN 1: Participants** | | | |
| --- | --- | --- | --- |
| **A. Risk of Bias** | | | |
| *Describe the sources of data and criteria for participant selection:*  Retrospective, observational cohort study using ED triage and registration data on all ED visits. Data was retrieved from the hospital computer system over a 2-year period.  Inclusion criteria: All ED patients. Exclusion criteria: Participants who left prior to a clinical decision, patients under 16 years of age. | | | |
|  | | Dev | Val |
| - 1. Were appropriate data sources used, e.g. cohort, RCT or nested case-control study data? | | Y | Y |
| - 1. Were all inclusions and exclusions of participants appropriate? | | Y | Y |
| **Risk of bias introduced by selection of participants** | **RISK:**  *(low/ high/ unclear)* | **L** | **L** |
| *Rationale of bias rating:* | | | |
| Appropriate cohort data sources used. Inclusive and clearly defined selection criteria. Exclusion criteria reported. Appropriate inclusion and exclusion of participants. | | | |
| **B. Applicability** | | | |
| *Describe included participants, setting and dates:*  All adult patients (≥16 years) visiting one of six “unscheduled care centers” in three hospitals in Northern Glasgow (three EDs, two medical Acute Assessment Units, and one Minor Injuries Unit) over a 2-year period, from March 21, 2010 to March 20, 2012. | | | |
| **Concern that the included participants and setting do not match the review question** | **CONCERN:**  *(low/ high/ unclear)* | **L** | **L** |
| *Rationale of applicability rating:* | | | |
| Participants and settings match review question. | | | |

| **DOMAIN 2: Predictors** | | | |
| --- | --- | --- | --- |
| **A. Risk of Bias** | | | |
| *List and describe predictors included in the final model, e.g. definition and timing of assessment:*  Dev: choice of predictor variables was all information collected during registration and triage that had a potential correlation with admission. This was 9 unreported variables, each with multiple values. Assessed at triage and registration.  Val: acuity category (Manchester Triaging System [MTS]), acuity score (National Early Warning Score [NEWS]), ambulance arrival, referral source, and admission within the last year, age. Assessed at triage and registration. | | | |
|  | | Dev | Val |
| - 1. Were predictors defined and assessed in a similar way for all participants? | | PY | Y |
| - 1. Were predictor assessments made without knowledge of outcome data? | | Y | Y |
| - 1. Are all predictors available at the time the model is intended to be used? | | PY | PY |
| **Risk of bias introduced by predictors or their assessment** | **RISK:**  *(low/ high/ unclear)* | **L** | **L** |
| *Rationale of bias rating:*  Dev: Predictors were not listed or defined, but were chosen because they were expected to potentially correlate with admission.  Val: Predictors required no manipulation (assessed as is). One predictor variable, “admission within the last year,” may not be available when the model is intended for use: if the patient has not previously attended the hospital the visit would not be on file. It is not stated if this information could be contributed to the model at the time of triage by patient report. | | | |
| **B. Applicability** | | | |
| Concern that the definition, assessment or timing of predictors in the model do not match the review question | **CONCERN:**  *(low/ high/ unclear)* | **L** | **L** |
| *Rationale of applicability rating:*  Dev: Although predictors were not reported, predictors were chosen that had a potential correlation with admission and were assessed at appropriate time. Thus, predictors, their assessment, and timing likely match review question.  Val: Predictor definitions, assessment, timing are clear and unmanipulated, and match review question. | | | |

| **DOMAIN 3: Outcome** | | | |
| --- | --- | --- | --- |
| **A. Risk of Bias** | | | |
| *Describe the outcome, how it was defined and determined, and the time interval between predictor assessment and outcome determination:*  Outcome: Patient final disposition defined by patient admission or discharge. Interval between predictor assessment was not reported and would vary per patient, patient condition, organizational processes, and facility capacity. | | | |
|  | | Dev | Val |
| - 1. Was the outcome determined appropriately? | | Y | Y |
| - 1. Was a pre-specified or standard outcome definition used? | | Y | Y |
| - 1. Were predictors excluded from the outcome definition? | | Y | Y |
| - 1. Was the outcome defined and determined in a similar way for all participants? | | Y | Y |
| - 1. Was the outcome determined without knowledge of predictor information? | | Y | Y |
| - 1. Was the time interval between predictor assessment and outcome determination appropriate? | | Y | Y |
| **Risk of bias introduced by the outcome or its determination** | **RISK:**  *(low/ high/ unclear)* | **L** | **L** |
| *Rationale of bias rating:*  Clearly defined outcome. | | | |
| **B. Applicability** | | | |
| *At what time point was the outcome determined:*  When the ED physician had enough information to determine that the patient required hospital admission.  *If a composite outcome was used, describe the relative frequency/distribution of each contributing outcome:*  n/a | | | |
| **Concern that the outcome, its definition, timing or determination do not match the review question** | **CONCERN:**  *(low/ high/ unclear)* | **L** | **L** |
| *Rationale of applicability rating:*  Timing of outcome determination appropriate to environment. | | | |

| **DOMAIN 4: Analysis** | | | |
| --- | --- | --- | --- |
| **Risk of Bias** | | | |
| *Describe numbers of participants, number of candidate predictors, outcome events and events per candidate predictor:*  Derivation group (n=215,231); validation group (n=107,615); 10 candidate predictors; 1 outcome event;  Events per candidate predictor (derivation): could not be calculated because *LSS unknown because % admitted or discharged patients not reported.* | | | |
| *Describe how the model was developed (for example in regards to modelling technique (e.g. survival or logistic modelling), predictor selection, and risk group definition):*  Initial variable inclusion was all variables in reception and triage that had a potential correlation to admission.  Logistic regression with stepwise deletion using a mixed effect model to account for patients who had multiple attendances. A final score was created by transforming the regression coefficients using normalization and rounding./Clinical variables that had significant associations with admission on logistic regression were entered into a mixed-effects multiple logistic model. This provided weightings for the score, which was then simplified and tested on a separate validation group by receiving operator characteristic (ROC) analysis and goodness-of-fit tests (not specified which tests). | | | |
| *Describe whether and how the model was validated, either internally (e.g. bootstrapping, cross validation, random split sample) or externally (e.g. temporal validation, geographical validation, different setting, different type of participants):*  Internal validation: samples across 6 sites were randomized and split | | | |
| *Describe the performance measures of the model, e.g. (re)calibration, discrimination, (re)classification, net benefit, and whether they were adjusted for optimism:*  AUROC and Goodness of fit. Overfitting accounted for with model comparison to the validation model. | | | |
| *Describe any participants who were excluded from the analysis:*  Participants with duplicate entries, cases with randomly missing data, and transfers between units were excluded. | | | |
| *Describe missing data on predictors and outcomes as well as methods used for missing data:*  Missing fields were inferred from other data (i.e., if gender was missing and name was Margaret). Where it could not be inferred, the attendance was excluded. Exceptions to this were made where data were missing for reasons that would strongly affect the probability of admission. For example, the sickest patients with the highest probability of admission sometimes bypassed triage due to the severity of their condition, thus initial observations and electronic triage data did not exist. In these cases, imputation of missing fields from matched cases was used to minimize bias. Cases with randomly missing data were excluded. | | | |
|  | | Dev | Val |
| - 1. Were there a reasonable number of participants with the outcome? | | Y | Y |
| - 1. Were continuous and categorical predictors handled appropriately? | | N | N |
| - 1. Were all enrolled participants included in the analysis? | | N | N |
| - 1. Were participants with missing data handled appropriately? | | Y | Y |
| - 1. Was selection of predictors based on univariable analysis avoided? | | Y |  |
| - 1. Were complexities in the data (e.g. censoring, competing risks, sampling of controls) accounted for appropriately? | | NI | NI |
| - 1. Were relevant model performance measures evaluated appropriately? | | PN | PN |
| - 1. Were model overfitting and optimism in model performance accounted for? | | Y |  |
| - 1. Do predictors and their assigned weights in the final model correspond to the results from multivariable analysis? | | Y |  |
| **Risk of bias introduced by the analysis** | **RISK:**  *(low/ high/ unclear)* | **L** | **L** |
| *Rationale of bias rating:*  Pros:  Did not select predictors with univariate analysis, employed multivariate analysis.  There were no competing risks.  Clear description of handling of missing data.  Appropriate methods for replacing missing data (imputation).  All participants not included: Description of total number of participants, number of included participants, and why participants excluded.  Performance measured by AUROC and goodness of fit. No calibration graph was included.  Overfitting accounted for with model comparison to the validation model.  Cons:  Categorization and dichotomization of predictors (age was grouped) is not a preferred method of analysis. | | | |

**Step 4: Overall assessment**

| Use the following tables to reach overall judgements about risk of bias and concerns regarding applicability of the prediction model evaluation (development and/or validation) across all assessed domains.  *Complete for each evaluation of a distinct model.*   \| **Reaching an overall judgement about risk of bias of the prediction model evaluation** \| \| \| --- \| --- \| \| **Low risk of bias** \| If all domains were rated low risk of bias.  If a prediction model was developed without any external validation, and it was rated as low risk of bias for all domains, consider downgrading to **high risk of bias**. Such a model can only be considered as low risk of bias, if the development was based on a very large data set and included some form of internal validation. \| \| **High risk of bias** \| If at least one domain is judged to be at **high risk of bias**. \| \| **Unclear risk of bias** \| If an unclear risk of bias was noted in at least one domain and it was low risk for all other domains. \|  \| **Reaching an overall judgement about applicability of the prediction model evaluation** \| \| \| --- \| --- \| \| **Low concerns regarding applicability** \| If low concerns regarding applicability for all domains, the prediction model evaluation is judged to have **low concerns regarding applicability**. \| \| **High concerns regarding applicability** \| If high concerns regarding applicability for at least one domain, the prediction model evaluation is judged to have **high concerns regarding applicability**. \| \| **Unclear concerns regarding applicability** \| If unclear concerns (but no “high concern”) regarding applicability for at least one domain, the prediction model evaluation is judged to have **unclear concerns regarding applicability** overall. \| |
| --- | --- | --- | --- | --- | --- | --- | --- | --- | --- | --- | --- | --- | --- | --- | --- | --- |

| **Overall judgement about risk of bias and applicability of the prediction model evaluation** | | |
| --- | --- | --- |
| **Overall judgement of risk of bias** | **RISK:**  *(low/ high/ unclear)* | **L** |
| *Summary of sources of potential bias:*  Categorization and dichotomization of predictors are not a preferred strategy because they cause loss of information that may be relevant, and the arbitrary data grouping changes the data, both can cause biases. | | |
| **Overall judgement of applicability** | **CONCERN:**  *(low/ high/ unclear)* | **L** |
| *Summary of applicability concerns:*  none | | |

1. Regression is the preferred technique for determining the statistical strength of the relationship between two or more predictor variables and a change in outcome, allows for predictor variables of multiple data types, and produces a mathematical equation for predicting the probability of the outcome occurring when only the predictors are known. [↑](#footnote-ref-1)
