# Supplementary material for: Models Predicting Hospital Admission of Adult Patients Utilizing Prehospital Data: Systematic Review Using PROBAST and CHARMS
Source: JMIR Med Inform. 2021 Sep 16;9(9):e30022. doi: 10.2196/30022 (PMC8485197; doi:10.2196/30022)
Supplement: Multimedia Appendix 3 [file medinform_v9i9e30022_app3.docx]

Multimedia Appendix 3. Predictors evaluated by each study.

| Predictors | References^a^ | | | | | | | | | | |
| --- | --- | --- | --- | --- | --- | --- | --- | --- | --- | --- | --- |
|  | [38] | [39] | [40] | [41] | [42] | [43] | [44] | [45] | [46] | [22] | [47] |
|  |  |  |  |  |  |  |  |  |  |  |  |
| Age | —^b^ | ✓ | — | ✓ | ✓ | ✓ | ✓  ≥60 years | ✓  ≥60 years | ✓ | ✓ | ✓ |
| Demographics | — | — | ✓ | — | — | — | — | — | — | — | — |
| Arrival mode | — | — | — | — | — | ✓ | — | — | ✓ | ✓ | — |
| Transport by personal, police, or walking | — | 🗶 | — | — | — | — | — | — | — | — | — |
| Ambulance arrival | — | ✓ | — | ✓ | — | — | — | — | — | — | ✓ |
| Arrival mode by own initiative, referral by general practitioner, ambulance, otherwise | — | — | — | — | ✓ | — | — | — | — | — | — |
| Walk-in arrival | — | — | — | — | — | — | — | — | — | — | 🗶 |
| Acuity measure | — | ✓ MTS^c^  ✓ NEWS^d^ | ✓ ESI^e^ | ✓ ATS^f^ | ✓ MTS/NTS^g^ | ✓ MTS | — | — | ✓ PACS^h^ | 🗶 ESI | ✓ PACS |
| Primary complaint | — | — | — | — | ✓ | ✓ | — | — | — | ✓ | — |
| Presenting symptom or diagnosis | — | — | — | ✓ | — | — | — | — | — | — | — |
| Specific complaint anywhere in record | — | — | — | — | — | — | 🗶 | — | — | — | — |
| Chest pain | — | — | — | — | — | — | ✓ | ✓ | — | — | — |
| Dizziness, weakness, or syncope | — | — | — | — | — | — | ✓ | ✓ | — | — | — |
| Dyspnea, shortness of breath | — | — | — | — | — | — | ✓ | ✓ | — | — | — |
| Diabetes | — | — | — | — | — | — | — | — | — | — | 🗶 |
| Hypertension | — | — | — | — | — | — | — | — | — | — | 🗶 |
| Dyslipidemia | — | — | — | — | — | — | — | — | — | — | 🗶 |
| Systolic blood pressure | ✓ ≤100 mmHg | — | — | — | — | ✓ | 🗶 | — | — | — | — |
| Pulse rate | ✓ ≥130 | — | — | — | — | ✓ | 🗶 | — | — | — | — |
| Respiration rate/O_2_ | ✓ ≥30 | — | — | — | — | ✓ | 🗶 | — | — | — | — |
| Temperature | ✓ 38.5 °C | — | — | — | — | ✓ | — | — | — | — | — |
| Fever status |  | — | — | — | — | — | — | — | ✓ | — | — |
| Impaired consciousness | ✓  AVPU^i^ | — | — | — | — | — | — | — | — |  | — |
| ED^j^ or fast track designation |  | — | — | — | — | — | — | — | — | ✓ | — |
| Referral source | — | ✓ | — | — | — | — | — | — | — | — | — |
| Outside referral | — |  | — | ✓ | — | — | — | — | — | — | — |
| Hospital admission within last year | — | ✓ | — | — | — | — | — | — | — | — | — |
| Medical history (categorized by organ system) | — | — | — | — | 🗶 | — | — | — | — | — | — |
| Hospital admission or ED visit in the preceding 3 months | — | — | — | — | — | — | — | — | — | — | 🗶 |
| Revisited ED within 30 days | — | — | — | — | — | ✓ | — | — | — | — | — |
| Number of ED visits in previous year | — | — | — | — | — | — | — | — | 🗶 | — | — |
| Hospital usage statistics | — | — | ✓ | — | — | — | — | — | — | — | — |
| History of diabetes | — | — | — | — | — | — | ✓ | ✓ | — | — | — |
| History of cancer | — | — | — | — | — | — | ✓ | ✓ | — | — | — |
| Past medical history | — | — | — | — | — | — | 🗶 | — | — | — | — |
| Sex | — | 🗶 | — | — | 🗶 | ✓ | 🗶 | — | 🗶 | — | 🗶 |
| Physician provider | — | — | — | — | — | — | — | — | — | 🗶 | — |
| Race | — | — | — | — | — | — | 🗶 | — | ✓ | — | — |
| Ethnic group | — | — | — | — | — | — | — | — | — | — | 🗶 |
| Number of home medications | — | — | — | — | — | — | 🗶 | — | — | — | — |
| Outpatient medication counts | — | — | ✓ | — | — | — | — | — | — | — | — |
| Time of day | — | 🗶 | — | — | — | — | — | — | ✓ | — | — |
| Triage time of day | — | — | — | ✓ | — | — | — | — | — | — | — |
| Day of week | — | — | — | ✓ | 🗶 | — | — | — | ✓ | — | — |
| Public school holiday | — | — | — | — | — | — | — | — | 🗶 | — | — |
| Lives alone | — | 🗶 | — | — | — | — | — | — | — | — | — |
| Service admitted to (surgery, medicine) | — | — | — | — | — | ✓ | — | — | — | — | — |
| Lab tests ordered | — | — | — | — | — | ✓ | — | — | — | — | — |
| Lab tests performed | — | — | — | — | 🗶 | — | — | — | — | — | — |
| Phlebotomised blood sample taken | — | — | — | — | — | ✓ | — | — | — | — | — |
| Radiology imaging performed | — | — | — | — | 🗶 | — | — | — | — | — | — |
| Postal code | — | — | — | — | — | — | — | — | ✓ | — | — |

^a^✓indicates that the predictor was retained in the final model; 🗶 indicates that the predictor was evaluated but not retained in the model.

^b^Predictor was not evaluated or included.

^c^MTS: Manchester Triage System.

^d^NEWS: National Early Warning Score.

^e^ESI: Emergency Severity Index.

^f^ATS: Australasian Triage Scale.

^g^NTS: National Triage Scale.

^h^PACS: Patient Acuity Category Scale.

^i^AVPU: Alert Verbal Pain Unresponsive Scale.

^j^ED: emergency department.
